# Success in the Dental Profession

**Published:** 1863-02

**Authors:** C. C. Dills

**Affiliations:** Piqua, O.


### SUCCESS IN THE DENTAL PROFESSION.

BY C. C. DILLS, D. D. S.

### No. 6.—Self-reliance; Confidence; Concentration.

"Trust thyself!" ———— ————"There is a time in every man's education when he arrives at the conviction that envy is ignorance; that imitation is suicide; that he must take himself for better, for worse, as his portion; that though the wide universe is full of good, no kernal of nourishing corn can come to him but through his toil bestowed on that plot of ground which is given to him to till."—*Emerson.*

I shall ever revere and uphold Ralph Waldo Emerson, if for nothing else than for the good his essay on Self-reliance has done me. Not, perhaps, that the tone of this article will show any lack of self-reliance on my part, but by nature I did possess it. I well remember with what awakening I read my first sentence in Emerson,—"There is one mind common to all individual men: Every man is an inlet to the same, and to all of the same: He that is once admitted to the right of reason is made freeman of the whole estate." Let me most earnestly recommend the above named essay to every self-distrusting soul,—that he consult it as his Bible, and flee to it as a city of refuge, in every fit of dispondency; and let him not be discouraged by its transcendentalism, but encouraged by its gracious sentiments. And again, for myself: although what I shall say in this article will be based on a sufficient experience to make it profitable, I may yet fail in the expression; for, however desirable it might be, that we should give our experience clearly in any particular, and thereby caution others against our errors, it can not be but that even one must have an experience for himself, to genuinely appreciate the good.

All professional men overrate the importance of their profession; (this is a shame, and I wish dentists would quit it;) and all writers overrate the importance of their subjects. But fortunately for me, I have not so far overrated the importance of my subjects heretofore, as to prevent my laying high claims, as elements of success, on self-reliance, confidence, and concentration. I do this on the very plain principle, that self-reliance is but a reliance on self,—the only one really to be depended upon; and that confidence in self is not only confidence in the one best calculated to act your peculiar part in life, but that one also most interested in you; and that this dealing with self is but staying at home, and attending one's own business,—is so concentrating ones forces that he can attend to that most successfully;—

> "Who bides at home, nor looks abroad,
> Carries the eagles, and masters the sword."

Let me be a little irregular in my consideration of these elements. Concentration as combining and centering many virtues, much force, is a power most potent and patient. It is the shot of the sharpshooter rather than that of the common soldier; it is the Rebel "Merrimac," rather than the Union "Cumberland;" it is the bombardment of Fort Sumpter, at the rate of two thousand balls and bomb-shells per day, rather than the blank-cartridge firing of a company of "Home-guards" on holiday parade; it is the policy of the Southern Army, rather than that of the Northern. Thus concentration effects. Plutarch asserts of the successful Pericles, that "There was, in the whole city, but one street in which he was ever seen, the street which led to the market place and the council-house." Wherever we see it, we can but note the success of concentration; but unfortunately we see more of the dissipation of its absence. Burns, the poet, attributed most of his misfortunes through life to the want of an aim. We are all familiar with individuals, capable of success, but failing in it by their disposition to change about, and the incident loss of strength in so doing. As poor Richard says,

> "I never saw an oft-removed tree,
> Not yet an oft-removed family,
> That throve so well as one that settled be."

Every thing these days is at war with self-trust, as well as with itself. Somehow we throw ourselves into the general whirl, and expect the crowd to save us; the government will work through, and I will be protected;—(forgetting that national strength originates in individual force, and is in exact ratio to it;)—the detector will tell me what is good, and I need not exert myself; some one else will do these things necessary for me, and yet which I dislike to do, and I can shun them. Sure enough we can, but not the penalty effixed the shunning. Every dental co-partnership is somewhat the result of self-distrust, and—as a penalty—lessens our capabilities, and possible earnings. The same is true of protective combinations generally, whether for a uniform fee-bill, or what not. I do not oppose combined effort, as such. I would only be selfish enough to be self-reliant. I would be independent until I was subjugated.—And I am not of the number disconcerted at the threatened mission of lucrative dentistry. As I believe in nature, so I do in her supreme rule, —in her laws of action and re-action, equalization and compensation, supply and demand. I could neither change them if I would, nor would if I could. "The gods sell all things at a fair price." What though vulcanite has been a hundred and fifty dollars per set, and to-day can be had for thirty?—Neither of these is the price of the gods!—When they settle on a fee-bill it will be right,—in the abstract, however discomforting in its immediate results,—*and they will settle upon one.* I am surprised that the intelligent business man is not fatalist enough to see how inevitably this thing of competition and prices works. " 'T is fine for us to speculate and elect our course, if we must accept an irresistible dictation."—

"The Destiny, minister general,
That executeth in this world over all,
The purveyance which God has seen before,
So strong it is, that tho' the world had sworn
The contrary of a thing by yea or no,
Yet sometimes it shall fallen on a day

That falleth not oft in a thousand years;
For, certainly, our appetites here,
Be it of war, or peace, or hate, or love,
All this is ruled by the sight above."

I believe California once petitioned Congress to protect her against the competition of foreigners in her gold mines, and, among other considerations, urged that the foreigner would send his gold home, and thereby lessen the amount of specie for our own country. Congress, apparently, acknowledged the principle as correct, until *Benton* very significantly suggested, that commerce usually regulates such things for herself,—that specie would ever go where there was the greatest demand for it. We all very well remember the bringing up of Mexican corn a few years ago, and very inconveniently experience the forty-five per cent premium for gold to-day. All this means something; there is a law deeper than the surface of things; the wise will acknowledge it, and try to harmonize themselves with it; the unwise can but kick against the pricks.

Some hero has said we should be willing to sacrifice this whole generation, in this inscrutible, yet I still trust God ordained war. I will not anticipate so sad a need, yet if it come I will acquiesce. Neither will I concern myself seriously for the future of the Dental Profession, much less for myself. If competition wax high, I will wax stronger, and still keep a stout heart. For though I know, many times "Providence has a wild, rough, incalculable road to its end, and will not mind to drown a man;" I will yet remember, that "we are now men, and must accept in the highest mind the same transcendent destiny, and not minors and invalids in a protected corner, nor cowards fleeing before a revolution." When I can no longer swim in this grand whirlpool of human action, I desire to, and should sink, a sacrifice to that Grander Circle

"Whose all prolific beam first called me forth
From darkness,—teeming darkness,—where I lay
The worm's inferior, and in rank beneath
The dust I thread on.——— ———"

Now, I would infuse a true courage into the breast of him I would influence. I would have him *accept* his life, for better for worse, as his portion. If a good providence prevail for us, why it shall be all well with us, and we should be religious enough to acknowledge it ;—and if a harsh fate, why we should so far accept that as to resist it with a fatal courage, or, at least, "draw resolution to meet it, from the impossibility of escaping it." "Why lads," said Napoleon to some raw recruits, "you must not fear death ; when soldiers brave death they drive him into the enemy's rank." A dauntless courage, self-reliance, confidence in self or a cause, or *determination of will,* has ever characterized great or successful men. It was this last trait that made Cæsar greater than the military world around him, and fitted Luther to lead the "insurrection of human thought against authority." It is said of the latter, that when advised not to go to a city notoriously thronged with his enemies, he replied, "Were there as many devils there as roof-tiles, I would on !" The principle holds good in private or business life. The old adage, that "Where there is a will there is a way," is no more common than true. If I talk with a man—but more especially with a woman—"Whose mouth grows wide, and face falls in," I fully expect him to become my patient ; I may be disappointed, but the feeling that he shall, enables me the better to perform my part, and tends to make him feel that he must. This influence of will upon will is all there is of mesmerism. Now, I trust no one will, for a moment, confound these elements of character I am advocating, with the vulgar traits of over-presumption, self-assurance, or self-conceit. Surely they are widely enough separated in fact. True self-reliance is a healthy *tone* of character, originating in intelligence, and existing in self-culture,—producing a knowledge of one's ability to do that assigned to him, and thus a true confidence. While, it is evident, the vulgar traits alluded to are founded and fostered in ignorance,—a *presumption* that all is right with self, and a consequent indisposition to self-culture.

Yet if I were asked which I would do, foster self-confidence, at the risk of self-conceit, or contemn self-conceit, at the expense of self-confidence, I answer, I would do the former. Not that I prefer impudence to reservedness, but that I would rather seek virtue in the surplus action of over confidence, than in the non-action of lack of confidence,—would rather trust the *effort* as of good result, than to trust, as good, no effort at all. In its application to dentistry, and general surgery, we are all perfectly familiar with the worth of confidence. Besides, the moral effect on self, of lack of confidence, attended, as it usually is, with a self-abnegation, is most lamentable, and tends, more than I know of, in a small way, to mar one's happiness, and make life and business both a failure.

Then trust yourself! Be assured that you not only *can* but *must* do for yourself, or pay a shameful tax for the luxury of inaction—*imbecility*. I look in vain for help outside of self. I only know my interests will be seen to when I look after them. If fortune has not already smiled on me, I know she is too whimsical to be depended upon in the future. And I know, too, how *perfectly futile* is all envy, covetousness, vain wishing for, perhaps, vainer estate, fretting, complaining, or writhing,—it but adds brands to the eternal burning. I know it is hard to preserve a sweet contentment inviolate, against the ever and anon presented poetical of an easy success, a big business, or, even rascally fortune, on the part of those around us, while we can but lay claim to success the most ordinary. But when we have the matter sounded correctly, and obtained that hard-earned mastery of self, fact, and circumstances lifting us above these formidable barriers of happiness, I know we must be contented still. I may not reasonably look for much happiness, as things are constituted, but I may reasonably look for more in either the cold contentment of the Laplander, or the colder acquiescence of the Stoic, than in the fatal fever of any discontent. "What can't be cured must be endured," is a saying worthy a philosopher; but let us *concentrate*, *stay at home*, *insist on self*, *depend* on self, and we will thus *prevent*, which is better than either to cure or endure. And thus may we secure most of the true pleasure and profit of life and business.

---

RETROSPECTIVE AND APOLOGETIC.

Rivarol has said, that "genius is only great patience," and another has suggested, that a manuscript be nine years old before its publication. These are valuable suggestions, but I have failed to appreciate, or appropriate them, else the series of articles which this closes, had not yet been commenced. I have been chagrined all along to see what a sacrifice of good heading for articles I was making, rather than to have been more patient. But genius I had not; and, I thought; odd, indeed, would appear articles nine years hence, written to-day, on any subject of progressive dentistry. The fact is, I commenced these articles by accident, and but irregularly saw the end from the beginning—that I would have so little favorable time to devote to them. But I will not apologize further. I have written, first, for my own pleasure and profit; second, for that of others, and certainly not at all for compositional effect. The first is commendable, as looking to self for benefit, the second, as looking to benefit others,—to please the publishers, discharge a duty, and sustain a good cause. Really, I feel it my serious duty to *think*, and to put my probable profitable thoughts on paper, *for dentistry*. As vary the autumnal tints, so do our minds; and as it takes all of human thought and action to constitute human life, so should be given each dentist's peculiar professional knowledge, to make perfect the dental profession. My share may be but a mite, and inferior; but it is consoling to know that the pages of the Register to-day are scarcely as valuable as were those of the Alexandrian or Vatican manuscripts in their days; and that it might be altogether proper to have in the Register a department for local, or articles of minor importance, as well as that for the highest professional knowledge, or knowledge from the highest professional dentists. For my part I no more want to see exclusive matter of fact in the Register, than I want exclusive contemplation of the real any where. (Eds. Register will excuse this liberty, and peculiar style of apology, of mine, for certainly I intend it more as encouragement for others to write than anything else.) To conclude: There have been in these articles some provoking blunders, either typographical or chirographical.

Piqua, O., Jan. 1863.

